# Exploring the Role of GLP‐1 Agents in Managing Diabetic Foot Ulcers: A Narrative and Systematic Review

**DOI:** 10.1111/wrr.70085

**Published:** 2025-09-01

**Authors:** Fiona S. Gruzmark, Gabriela E. Beraja, Ivan Jozic, Hadar A. Lev‐Tov

**Affiliations:** ^1^ Dr. Phillip Frost Department of Dermatology and Cutaneous Surgery University of Miami Miller School of Medicine Miami Florida USA

**Keywords:** amputation, diabetic foot ulcers, GLP‐1, systematic review, wound healing, wound repair

## Abstract

Globally, there are 537 million people with diabetes, with an estimated 19%–344% of these people developing a diabetic foot ulcer, and 10% dying within a year of being diagnosed with a diabetic foot ulcer. Risk factors for developing a diabetic foot ulcer include age, sex, ethnicity, chronically elevated HbA_1c_, smoking history, cardiovascular disease, end‐stage renal disease, and retinopathy. Diabetic foot ulcer recurrence rates are as high as 20%, and they have vast complications, including lower‐extremity amputations. More recently, there has been a surge in the use of glucagon‐like peptide 1 receptor agonists in managing diabetes and weight loss. The use of glucagon‐like peptide 1 receptor agonists in treating diabetic foot ulcers in humans has not been extensively studied, but there are reports of using glucagon‐like peptide 1 receptor agonists in other dermatologic diseases with positive outcomes, including androgenetic alopecia and hidradenitis suppurativa. This review aims to explore the potential of using systemic glucagon‐like peptide 1 receptor agonists in managing diabetic foot ulcers, describing their effects on modulating wound repair, microvascular function, neuropathic symptoms, apoptosis, weight loss, oxidative stress, and inflammation. Additionally, a systematic review, following PRISMA guidelines, was conducted assessing the rate of diabetic foot complications in patients using glucagon‐like peptide 1 receptor agonists when compared to a control group, with the results suggesting their potentially protective role. By managing multiple facets of diabetic foot ulcer pathophysiology, the use of glucagon‐like peptide 1 receptor agonists may aid in their management and thus prevent recurrence.

**Abbreviations**
11β‐HSD1: 11β‐hydroxysteroid dehydrogenase type 1
AGEs: advanced glycation end‐products
AMPK: AMP‐activated protein kinase
ATP: adenosine triphosphate
Bak: Bax and Bcl‐2 homologous antagonist/killer
Bax: BCL2 associated X
Bcl‐2: B‐cell leukaemia/lymphoma 2 protein
CAMP: cyclic AMP
DFC: diabetic foot complication
DFU: diabetic foot ulcer
DM: diabetes mellitus
ERK 1/2: extracellular signal‐regulated protein kinases 1 and 2
GIP: gastric inhibitory polypeptide
GLP‐1RAs: glucagon like peptide 1 receptor agonists
HDAC6: histone deacetylase 6
hsCRP: high‐sensitivity C‐reactive protein
IFN‐γ: interferon gamma
IL‐10: interleukin‐10
IL‐17: interleukin‐17
IL‐1β: interleukin‐1β
IL‐2: interleukin‐2
IL‐6: interleukin‐6
LEA: lower‐extremity amputations
MCP‐1: monocyte chemoattractant protein‐1
NF‐kB: nuclear factor kappa B
NO: nitric oxide
NRF2: nuclear factor erythroid 2‐related factor
eNOS: endothelial nitric oxide synthase
PAD: peripheral arterial disease
PGC‐1α: peroxisome proliferator‐activated receptor gamma coactivator 1α
PKC: protein kinase C
RAGE: receptor advanced glycation end‐products
RCT: randomized control trial
ROS: reactive oxygen species
STAT3: signal transducer and activator of transcription 3
TEWL: transepidermal water loss
TLR‐2: toll‐like receptor 2
TLR‐4: toll‐like receptor 4
T1DM: type 1 diabetes mellitus
T2DM: type 2 diabetes mellitus
TNF‐α: tumour necrosis factor alpha
UCP2: uncoupling protein 2
VEGF: vascular endothelial growth factor

## 1 Introduction

Diabetic foot ulcers (DFUs) are defined as a foot ulcer in those, either currently or previously, diagnosed with diabetes mellitus and typically have peripheral neuropathy or peripheral arterial disease of the lower extremity [1]. DFUs are characterized as a disturbance of the epidermis and at least partial dermis, often being caused by repetitive trauma to the feet from increased pressure at specific plantar sites, poorly fitting shoes, abnormal gaits, and/or injuries [2]. There are over 500 million people with diabetes worldwide [3], and it is estimated that 19%–34% of these people will develop a DFU [4], with 20% of those with DFUs requiring lower‐extremity amputations (LEA) [4] and 10% dying within a year of diagnosis [5]. With a global prevalence rate of 6.3% [6], a recurrence rate over 20% [7] and a 10‐year mortality rate of 76.9% [8], DFUs pose a substantial burden to the healthcare system [9, 10]. They also pose an economic burden, with diabetes care accounting for $273 billion in direct costs and $90 billion in indirect costs annually [11], with foot complications exceeding 50%–200% above the baseline cost of care [12].

The aetiology of DFUs can be characterized as neuropathic, ischaemic, or neuroischaemic, depending on the presence of peripheral neuropathy and peripheral arterial disease (PAD) [2]. The hyperglycaemia observed in diabetes leads to oxidative stress while also activating signalling pathways that activate apoptosis, culminating in deregulated inflammatory responses and aberrant keratinocyte function [13]. Some risks of developing a DFU include increasing age, sex (male), race (Black, Hispanic or non‐White), chronically elevated HbA_1c_, a history of smoking, cardiovascular disease, end‐stage renal disease, and retinopathy [2].

The current standard treatment for DFUs includes debriding necrotic tissue, off‐loading the wound, appropriately managing blood glucose (with a goal HbA_1c_ less than 8%), managing infection, evaluating for PAD to determine if revascularization is necessary, and using appropriate wound dressings [14, 15]. Preventative measures include regular foot exams (to assess for neuropathy, foot deformities, joint mobility, calluses and pre‐ulcerative signs), wearing protective or orthotic footwear and daily washing of the feet followed by emollient use [16]. However, DFUs remain a challenging complication of diabetes, with high mortality and recurrence rates, largely because of ineffective treatment regimens that currently exist for managing DFUs [8].

Glucagon‐like peptide‐1 (GLP‐1) receptor agonists (GLP‐1RAs) have recently gained popularity in treating diabetes [17]. GLP‐1 s are insulinotropic agents in patients with type 2 diabetes mellitus (T2DM) that reduce plasma glucose, as well as glucagon secretion, slow gastric emptying, while also reducing caloric intake [18]. There are various GLP‐1RAs approved for treating diabetes, including dulaglutide, exenatide, liraglutide, lixisenatide, semaglutide, and albiglutide [19, 20] (Table 1). The most common side effects of these agents include nausea, vomiting, and diarrhoea [20].

**TABLE 1:** Recommended dosing regimens for glucagon like peptide‐1 receptor agonists approved for diabetes [18, 19, 20].

Glucagon like peptide‐1 Receptor agonist	Route	Dosing regimen
Dulaglutide^a^	SC	Start at 0.75 mg SC once weekly. Titrate to 1.5 mg SC once weekly or maximally tolerated dose.
Exenatide (twice daily)	SC	Start at 5 mcg SC twice daily. Titrate to 10 mcg SC twice daily after one month.
Exenatide (ER)	SC	2 mg SC once weekly
Liraglutide^a^	SC	Start at 0.6 mg SC daily once weekly. Titrate to 1.2 mg SC daily, and further to 1.8 mg SC daily if needed.
Lixisenatide	SC	Start at 10 mcg SC daily for 2 weeks. Increase to 20 mcg SC daily.
Semaglutide^a^	SC	Start at 0.25 mg SC once weekly for four weeks. Increase to 0.5 mg SC once weekly, and further to 1 mg SC once weekly if needed.
	Oral	Start at 3 mg oral daily for 30 days. Increase to 7 mg oral daily, and further to 14 mg oral daily if needed
Albiglutide^b^	SC	30 mg SC once weekly. Increase to 50 mg if needed

Abbreviations: ER, extended release; SC, subcutaneous.

^a^ Preferred agents based on reducing major adverse cardiovascular events.

^b^ Not available in the United States.

**FIGURE 1:**
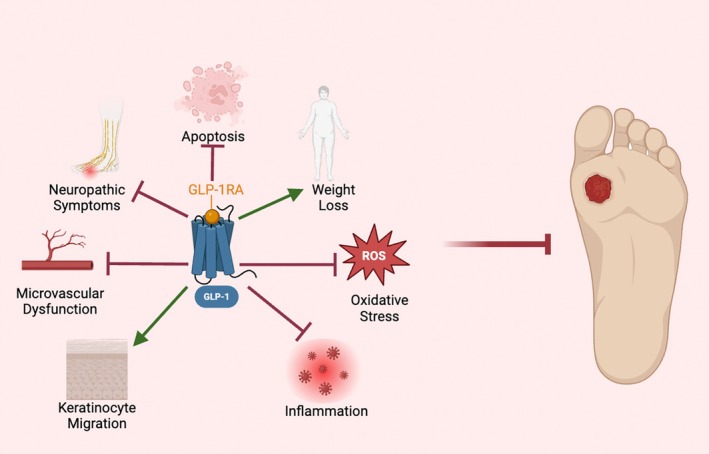
Mechanisms GLP‐1RAs Modulate that Mediate DFUs. This figure illustrates the mechanisms of DFU development that GLP‐1RAs modulate to prevent DFU development.

Although the use of GLP‐1RAs in treating DFUs in humans has not been extensively studied, there are some studies suggesting GLP‐1RAs utility in DFU management. For instance, one cohort study found that in patients with T2DM, those using GLP‐1RAs had improved DFU wound healing when compared to those on insulin [21]. Additionally, there have been numerous reviews discussing the effect of GLP‐1RAs on different body systems [22], with some reports suggesting the use of GLP‐1RAs in the treatment of androgenic alopecia [23], psoriasis [22], as well as hidradenitis suppurativa [21], all suggesting an improvement in disease severity and quality of life. These findings highlight the potential of GLP‐1RAs in modulating the skin, suggesting they may offer therapeutic benefits beyond their current indications and warrant further exploration in the treatment of DFUs as adjunctive therapies. This review aims to decipher the potential of using systemic GLP‐1RAs in managing DFUs by shedding evidence on their multifactorial benefits via modulation of wound repair, microvascular function, neuropathic symptoms, apoptosis, weight loss, oxidative stress, and inflammation (Figure 1). Additionally, a systematic review was conducted assessing the rate of diabetic foot complications in patients using GLP‐1RAs.

## 2 The Pathophysiology of Skin Changes in Diabetes Mellitus

Chronic hyperglycemia in diabetes mellitus (DM) triggers a cascade of biochemical and physiological changes that profoundly impact the skin. One key mechanism involves the localized skin‐specific activation of 11β‐hydroxysteroid dehydrogenase type 1 (11β‐HSD1) [24], which converts inactive cortisone into active cortisol. Elevated cortisol levels induce endoplasmic reticulum stress, impairing skin barrier function by depleting essential lipids and reducing stratum corneum hydration, leading to a compromized and dysfunctional barrier [25, 26]. Dysregulated 11β‐HSD1 expression inhibits fibroblast proliferation and collagen synthesis, keratinocyte migration, together leading to impaired wound healing [24, 27]. In db/db diabetic mice, significantly higher 11β‐HSD1 expression elevates endogenous glucocorticoid levels, reducing skin repair capacity and highlighting its role in delayed healing associated with diabetes [24, 28]. Wound treatment with 11β‐HSD1 inhibition has demonstrated efficacy in mouse skin, particularly in ob/ob mice, and 11β‐HSD1 knockout mice are protected from the adverse effects of excess circulating endogenous glucocorticoid levels [29, 30, 31, 32]. A double‐blind, randomized, placebo‐controlled pilot trial was conducted using punch biopsies from individuals with T2DM who received 400 mg of the oral 11β‐HSD1 inhibitor, AZD4017, demonstrating faster healing rates and improved skin integrity [33], ultimately suggesting that 11β‐HSD1 inhibitors may be a novel therapy for DFUs.

Hyperglycemia also promotes the formation of advanced glycation end‐products (AGEs), which are a normal part of aging when reducing carbohydrates react with proteins and lipids. When AGEs bind to their receptor (RAGE), they cross‐link proteins and disrupt important cell signalling pathways, leading to dysregulated apoptosis and may aberrantly affect physiological processes like wound healing [34]. AGEs also impair collagen function and reduce growth factor proliferation, such as fibroblast growth factor, leading to decreased skin elasticity, contractility, and increased stiffness [35]. Goova et al. demonstrated that blocking RAGE with soluble (s)RAGE in diabetic mice improved wound healing by shortening the inflammatory phase, reducing metalloproteinase activity, and promoting faster repair [36]. Accumulation of AGEs has also been demonstrated to activate the nuclear factor kappa B (NF‐kB) pathway and, subsequently, increase expression of inflammatory cytokines, thus perpetuating the inflammatory response noted in diabetic‐related wounds [37]. Reactive oxygen species (ROS) and nitric oxide (NO) levels have been reported to increase as AGE levels rise [38, 39]. AGEs impair fibroblast and endothelial cell function, reducing proliferation, migration, and granulation tissue formation, leading to chronic wounds and delayed skin repair [40].

As previously alluded to, DM also compromises the skin's structural integrity and hydration [9]. A case–control study demonstrated reduced transepidermal water loss (TEWL), stratum corneum hydration, and stratum corneum lipid levels in individuals with T2DM, reflecting significant skin barrier dysfunction [26]. Furthermore, stratum corneum hydration has also been demonstrated to negatively correlate with HbA1c levels, thus linking poor glycemic control to impaired skin health [26]. Similarly, in a population‐based study of 3190 adults, elevated HbA1c levels were associated with microvascular complications, including peripheral neuropathy, which exacerbates skin breakdown and delays wound healing [41].

Microvascular dysfunction plays a critical role in the progression of diabetic skin complications. Pericytes are contractile cells that interact with a milieu of cells in the wound microenvironment (endothelial/epithelial fibroblasts and leukocytes), and act to regulate neovascularization during wound healing by regulating vessel formation and stabilization [42, 43]. In a diabetic mouse model, Okonkwo et al. demonstrated that skin wounds in db/db diabetic mice exhibited reduced pericyte coverage and increased capillary permeability, along with significantly lower expression of pro‐angiogenic factors and those responsible for pericyte recruitment and vessel maturation, thus reducing the efficiency of nutrient and oxygen delivery to the skin [44].

An important factor in diabetic foot ulcer (DFU) healing is the unique physiology of plantar skin, given the tendency of DFUs to develop on plantar surfaces. Volar skin surfaces lack hair and sebaceous glands, have increased epidermal turnover, thickened stratum corneum, unique keratin filament structures, and less permeable barriers than non‐volar skin. One study, using porcine models, demonstrated that volar skin has unique molecular, gene, and protein phenotypes resembling wounded skin. For instance, plantar epidermis was found to have keratinocyte hypertrophy, spongiosis, a flattened Ca2+ gradient, ST1M1 downregulation, S100 upregulation, persistent K14 in the suprabasal epidermis, K10 reduction, and stress keratin expression (K6, K16, K17). Stress keratins increase keratinocyte mechanical resilience in these high‐pressure areas of volar skin, but they also delay epithelialization. Additionally, this study demonstrated that in porcine models, wounded volar skin that was both weight‐bearing and non‐weight‐bearing took significantly longer to close when compared to dorsal foot wounds, with delayed contraction and re‐epithelialization [45].

## 3 GLP‐1 Agonists' Mechanism of Action and Systemic Effects

### 3.1 Mechanism of Action and Differences Amongst GLP‐1RAs

GLP‐1RAs act as an incretin hormone, inhibiting glucagon secretion, promoting weight loss, and reducing inflammation [46]. As previously mentioned, there are various GLP‐1RAs that are approved for treating diabetes, including short‐acting (exenatide and lixisenatide) and long‐acting (liraglutide, dulaglutide, semaglutide and albiglutide). Moreover, tirzepatide is a dual GLP‐1RA/gastric inhibitory polypeptide (GIP) agonist with demonstrated efficacy in the treatment of diabetes. Interestingly, these agents have also demonstrated efficacy in achieving weight loss, regardless of whether the patient has diabetes or not. In a cohort study of 41,222 overweight or obese patients receiving either semaglutide or tirzepatide, patients receiving tirzepatide were more likely to achieve weight loss, with no significant differences in gastrointestinal adverse events [47]. In an adjusted indirect treatment comparison study assessing the efficacy of semaglutide in comparison to tirzepatide in patients with T2DM, both 10 and 15 mg tirzepatide exhibited significantly reduced HbA_1c_ and achieved greater weight loss than semaglutide 2 mg [48]. These results potentially suggest that dual GLP‐1/GIP receptor agonists could perform better than GLP‐1RA in head‐to‐head clinical trials assessing weight loss and HbA_1c_. Also important to note is that retatrutide, a triple GIP, GLP‐1, and glucagon receptor agonist, also shows promising results. In a Phase 1b double‐blind placebo‐controlled randomized controlled trial (RCT) of patients with T2DM, retatrutide was found to more effectively reduce plasma glucose, HbA_1c_ and weight when compared to placebo and liraglutide [49]. Altogether, these findings highlight the importance of tailoring GLP‐1RA selection based on individual patients' goals, as emerging therapies, including dual and triple receptor agonists, show potential not only for weight loss and glycemic control but also for further exploration in wound healing.

### 3.2 Microvascular Function

It has been well‐reported that microangiopathy is an important mediator of DFUs, with hyperglycemia being a causative factor, due to its disruption of vascular tone, vascular permeability and auto‐regulation of blood flow, leading to disrupted endoneurial perfusion and hypoxia, subsequently altering cellular metabolism and impairing wound healing [50]. These changes suggest that controlling microvascular disruption may help mediate DFU formation. Importantly, several studies have demonstrated the effect of GLP‐1RAs on microvascular function. In a study assessing the use of exenatide in murine models with modified dorsal McFarlane flaps, exenatide increased flap survival and microvascular density, while also increasing vascular endothelial growth factor (VEGF) expression, and reducing inflammatory cytokine expression of Interleukin‐6 (IL‐6), Interleukin‐1β (IL‐1β), NF‐κB, toll‐like receptor 4 (TLR4), and tumour necrosis factor alpha (TNF‐α) [51]. Although this study did not assess microvascular function specifically in DFUs, this demonstrated potential that increased microvascular density may help reduce the hypoxia seen in DFUs. Moreover, patients with DFUs have been shown to exhibit elevated plasma levels of IL‐6 and TNF‐α [52], and wound exudate levels of IL‐1β, NF‐κB and TLR4 [53], suggesting the beneficial anti‐inflammatory role that exenatide can have in DFUs. In another murine study assessing the effect of liraglutide on diabetic wounds, liraglutide was found to promote wound healing by enhancing angiogenesis, regulated by the HIF‐1a‐heme oxygenase‐1 axis, and by regulating AMP‐activated protein kinase (AMPK) for endothelial promotion [54].

Importantly, vasculature expresses GLP‐1Rs, and GLP‐1 has been shown to improve endothelial function and microvascular perfusion [55]. In a RCT assessing the effect of liraglutide and exenatide in patients with and without diabetes, injection of both liraglutide and exenatide increased local skin perfusion when compared to saline, in a mechanism thought to be mediated by endothelial nitric oxide synthase (eNOS) [56]. This study, while specific to patients with T2DM and not DFUs, shows promise in altering the dysfunctional microvasculature in patients with T2DM, which may help to address DFU development.

### 3.3 Neuropathic Symptoms

Another important mediator of DFU development is neuropathic symptoms, which are due to damage from hyperglycemia, and its relation to adenosine triphosphate (ATP) deficiency [52], the polyol pathway [57], oxidative stress [58], protein kinase C (PKC) activation, and a proinflammatory state [59]. First, decreased ATP leads to reduced axonal transport, as well as an inability to counteract oxidative stress [60]. Second, in the polyol pathway, hyperglycemia causes elevated sorbitol levels, which reduce sodium–potassium ATPase activity, reducing conduction velocities while also inducing oxidative stress. Lastly, PKC is involved in microvascular dysfunction, causing impaired blood flow to peripheral nerves, since hyperglycemia activates PKC [61], and activated PKC limits intracellular calcium levels involved in calcium signalling pathways of α_1_‐adrenoceptor in resistance arteries [62]. Together, these mechanisms cause sensory neuropathies that impair protective sensations, causing trauma and unnoticed injuries. Additionally, motor neuropathies cause atrophy and mispositioned feet, increasing plantar pressures. Autonomic neuropathies cause dysfunctional sweat and sebaceous glands, causing fissures in the skin. Altogether, these impairments promote DFUs [60].

There are mixed studies supporting the role that GLP‐1RAs play in mediating peripheral neuropathy. In one RCT assessing the effect of exenatide compared to insulin glargine in adult patients with T2DM, there was no difference in the peripheral neuropathy experienced in patients between these two groups, and exenatide did not reduce the prevalence of peripheral neuropathy, either [63]. In a separate RCT assessing diabetic neuropathy in patients with type 1 diabetes (T1DM), liraglutide was not shown to alter neuronal function; however, inflammatory cytokines like IL‐6 were reduced, which may modulate the neuroinflammatory component of peripheral neuropathy [64]. Interestingly, in a RCT assessing corneal regeneration in patients with T2DM, treatment with exenatide and pioglitazone promoted corneal nerve regeneration [65]. In a post hoc analysis of a RCT assessing the role of liraglutide in adult patients with T1DM, liraglutide was found to have the potential of improving enteric nervous system functioning [66]. Other animal studies have also supported the neuroprotective role of GLP‐1RAs [67], suggesting the role GLP‐1RAs may play in preventing neurodegeneration, and therefore, DFU formation.

### 3.4 Apoptosis

It has been well‐reported that programmed cell death, including apoptosis, autophagy, ferroptosis, and pyroptosis, is all involved in the pathogenesis of DFUs [68]. In diabetic wounds, the macrophages, endothelial cells, and keratinocytes involved in the healing process undergo abnormal apoptosis, causing abnormal angiogenesis, inflammation, and re‐epithelialization. Granulation tissue remodelling is also associated with fibroblast apoptosis in diabetic wounds. These abnormalities may be promoted by the AGEs seen in diabetes. Elevated TNF‐α levels in diabetes also activate FOXO1, which regulates apoptosis [69]. Additionally, the transcription factors FOXM1 and signal transducer and activator of transcription 3 (STAT3), transcription factors functioning to promote immune cell survival, are inhibited in DFUs. In diabetic mouse models, FOXM1 inhibition has been shown to delay wound healing [10]. This suggests that mediating apoptosis can help manage DFUs.

It has been reported that in murine studies, GLP‐1RAs exhibit reduced apoptosis in various cell types. In a study assessing apoptosis of hepatocytes in hyperglycaemic conditions treated with GLP‐1, BCL2 associated X (Bax) and B‐cell leukaemia/lymphoma 2 protein (Bcl‐2) homologous antagonist/killer (Bak) levels increased when exposed to GLP‐1, miR‐23a expression levels were attenuated with GLP‐1 exposure, Peroxisome proliferator‐activated receptor gamma coactivator 1α (PGC‐1α) mRNA expression increased after GLP‐1 exposure, and Uncoupling protein 2 (UCP2) expression increased after GLP‐1 exposure [70]. These findings suggest that GLP‐1 induced PGC‐1α expression by down‐regulating miR‐23a, which inhibited hepatocyte apoptosis while enhancing UCP2 expression to reduce apoptosis. PGC‐1α is a mitochondrial protective gene and UCP2 is a protein that functions to protect mitochondria from apoptosis and oxidative stress, while miR‐23a has been shown to inhibit mitochondrial synthesis [70]. In another mouse study assessing the effects of hyperglycaemia on renal tubular cells, there was greater cell viability when treated with liraglutide, with these effects reversed by a GLP‐1R antagonist [71]. These anti‐apoptotic effects of GLP‐1RA in hyperglycaemic states have also been observed in pancreatic islet cells [72] and cardiomyocytes [73]. Although the effects of cellular apoptosis by GLP‐1RAs of cells mediating wound healing have not been studied, the aforementioned studies regarding apoptosis suggest its promise in reducing apoptosis in cells contributing to DFUs.

In a study assessing the effect of exendin‐4 in addressing mitochondrial and oxidative impairments in neuroblastoma cells, exendin‐4 was found to increase expression of phospho‐Akt/Akt and Bcl‐2, which are cell survival markers. Exendin‐4 was also found to decrease Bax expression, a pro‐apoptotic marker. There were also lower levels of catalase, superoxide dismutase 2, and heme oxygenase 1, which are ROS defence markers. Additionally, there were lower expression levels of mitochondrial function associated genes, MCU and UCP3, and mitochondrial fission genes, DRP1 and FIS1, when treated with exendin‐4. Expression levels of Parkin and PINK1, regulators of mitochondrial homeostasis, were also increased. Importantly, defective mitochondria accumulate PINK1, and decreased Parkin levels are seen in diabetes, with dysregulated Parkin playing a key factor in diabetic neurodegeneration. Altered mitophagy is also seen to play a role in diabetic neuropathy. This data suggests that GLP‐1RAs may play a neuroprotective role in diabetic patients by mediating mitochondrial dysfunction [74]. Altogether, GLP‐1RAs may mediate DFU pathogenesis by modulating apoptosis.

### 3.5 Weight Loss

In a systematic review evaluating risk factors for developing DFUs from 3 cohort studies, 22 case‐control studies, and one cross‐sectional study, obesity was found to be a significant risk factor [75], which suggests that weight loss in some patients with diabetes may help prevent DFU development. In fact, weight loss is recommended in diabetic patients not only for glycemic control, but also to reduce the pressure load on the foot, which has been demonstrated to predispose these patients to the development of DFUs [76]. As alluded to previously, GLP‐1RAs have been found effective for managing patients' weight. In a phase 3 RCT of 1595 patients who were overweight or obese and had diabetes, semaglutide 2.4 mg injected weekly was found to be superior in achieving a decrease in bodyweight when compared to semaglutide 1.0 mg and placebo [77]. GLP‐1RAs currently approved by the FDA to treat overweight and obesity include liraglutide, semaglutide and tirzepatide [78]. Ultimately, weight loss in patients who are overweight or obese can help prevent or manage DFU development and progression, suggesting the role of GLP‐1RAs being implemented into the treatment regimen of DFUs.

### 3.6 Oxidative Stress

Oxidative stress plays a significant role in DFU development, as hyperglycemia in diabetic patients causes mitochondrial ROS development, lipid peroxidation, macromolecule destruction, and delayed wound healing. Molecular mechanisms implicated in the pathogenesis of DFU development include the diacylglycerol pathway [79], hexose pathway [80], NO pathway [81], polyol pathway [57], PKC pathway [59] and AGEs [80], which are all mechanisms that are caused by mitochondrial ROSs. Conventionally, nuclear factor erythroid 2‐related factor (NRF2) is activated during oxidative stress to reduce apoptosis and encourage wound healing [82]. Importantly, levels of oxidative stress caused by diabetes can be reduced by NRF2 activation, which regulates matrix metalloproteinase 9 and TGF‐β, mediating cellular migration and proliferation [83]. Prolonged wound healing is also promoted by activating transcription factor 3, inducible nitric oxide synthase, caspase −3, −8, and −9 [82]. Notably, there are numerous molecular pathways that encourage delayed wound healing in DFUs secondary to oxidative stress.

There is some evidence to suggest that GLP‐1RAs mediate the oxidative stress that accompanies DFUs. One study found that liraglutide reduced the oxidative stress induced by methylglyoxal, increased SOD activity, decreased ROS content, and increased *P22phox*, *Gp91phox*, and *Xdh* gene expression levels in SH‐5Y5Y cells [84]. Methylglyoxal is a precursor of AGEs and is elevated in patients with diabetes, while also being implicated in promoting oxidative stress, mitochondrial dysfunction, and metabolic disorders. SOD is an antioxidant that limits lipid peroxidation. *P22phox*, *Gp91phox*, and *Xdh* genes code for ROS‐producing enzymes [84]. Another study found that in human umbilical vein endothelial cells exposed to oxidative stress, GLP‐1 treatment reduced the oxidative stress on these cells by restoring histone deacetylase 6 (HDAC6) expression by attenuating ERK1/2 phosphorylation. Importantly, HDAC6 mediates increased cell migration and decreased autophagy [85]. Although these studies did not evaluate the role of GLP‐1RAs in mediating oxidative stress in DFUs, their role in reducing oxidative stress in other cellular pathways suggests their usefulness in managing DFU development.

### 3.7 Inflammation

An abnormal inflammatory response plays a key role in the pathogenesis of DFUs and delayed wound healing, characterized by increased expression of cellular inflammatory protein factors, decreased levels of β‐Defensin, abnormal activation of sensor activation signal and transcription activator, decreased activity of NF‐κB, decreased protein kinase B expression, and macrophage polarization [82, 86, 87, 88, 89, 90]. Patients with diabetes, both with and without complications, also have higher serology levels of interleukin‐17 (IL‐17) [91]. When looking at transcriptomic data sets, patients with DFUs had higher levels of IL‐1β expression [92]. Additionally, when looking at circulating levels of TNF‐α in patients with diabetes, patients with DFUs had higher levels of TNF‐α, high‐sensitivity C‐reactive protein (hsCRP) and IL‐6 compared to those without a DFU [93]. In vivo mice models and in vitro human skin, DFU wounds had higher levels of TLR‐4 [94]. Another study showed that mice with diabetes have higher levels of toll‐like receptor 2 (TLR‐2) when compared to mice without diabetes when measuring TLR‐2 mRNA and protein expression, and increased TLR‐2 was found to inhibit wound healing [95]. Notably, keratinocytes that were cultured in a high glucose environment had mRNA expression of interleukin‐8 1.5 times higher than in a normal glucose environment [96], suggesting that keratinocytes in patients with diabetes promote a pro‐inflammatory environment, increasing interleukin‐8 expression, which elevates ROS levels [97]. Ultimately, regulating the increased expression of pro‐inflammatory cytokines and abnormally functioning macrophages and neutrophils can help prevent DFU development [82].

There is evidence that suggests GLP‐1RAs regulate inflammatory responses, which may suggest their potential role in managing DFUs. Importantly, exendin and exenatide have been found to suppress interferon gamma (IFN‐γ), IL‐17, interleukin‐2 (IL‐2) and IL‐1β expression in vitro human islets [98], suppressed IL‐1β expression in vitro β‐cell assays of humans, mice, and rats [99], and decreased TNF‐α and IL‐1β in LPS‐treated human monocytes/macrophages [100]. Exenatide has also been shown to change macrophages to their anti‐inflammatory phenotype in cultured human monocytes/macrophages [100]. GLP‐1 exposure increased interleukin‐10 (IL‐10) levels and activated the STAT3 pathway in human cultured macrophages [101] and cultured macrophages from apolipoprotein E null mice [102], which are both anti‐inflammatory responses [103]. In a clinical trial of 24 patients with T2DM and obesity injected with exenatide 10 μg twice daily, exenatide was found to reduce TNF‐α, TLR‐2, TLR‐4, IL‐1β, monocyte chemoattractant protein‐1 (MCP‐1) and IL‐6 [103, 104]. In clinical trials of patients who were overweight or obese and had T2DM, liraglutide was found to reduce IL‐6, TNF‐α, sCD163, IL‐1β and IL‐6 [103, 105, 106, 107]. In clinical trials of patients who had T2DM or were obese, semaglutide was found to decrease hsCRP levels [103, 108, 109]. Notably, although not assessing inflammation in patients with diabetes, in a systematic review assessing the role of GLP‐1RAs in patients with HS, GLP‐1RAs were found to reduce TNF‐α, IL‐17, and NF‐κB levels in patients [23]. These findings suggest that GLP‐1RAs have a powerful anti‐inflammatory role, suggesting their utility in managing DFUs.

### 3.8 Keratinocyte Migration

Abnormal keratinocyte migration is another crucial mediator of DFU development [24, 110]. The exact mechanism leading to abnormal keratinocyte migration in DFUs is not known; however, it has been suggested that increased pro‐apoptotic bcl2 and caspase 3, suppressor of cytokine signalling 3, reduced keratin expression, decreased phosphorylated focal adhesion kinase, and increased non‐enzymatic glycation of type I collagen may be involved [97, 111, 112, 113, 114]. It has also been elucidated that in vitro mice models, FOXO1 deletion in high glucose media conditions did not impact TGFβ1 expression, indicating that FOXO1 does not interact with the TGFβ1 promoter in hyperglycemic conditions, which impairs keratinocyte migration. In standard glucose media, FOXO1 deletion in vitro reduced TGFβ1 levels [97, 115]. Additionally, when testing wound samples for gene expression, it has been shown that DFUs have higher levels of caveolin‐1, and caveolin‐1 impairs actin‐cytoskeletal signalling and keratinocyte migration [24].

There are various studies assessing the effectiveness of GLP‐1RAs in altering keratinocyte functionality. In one study, in which HaCat cells were incubated with LPS, liraglutide was able to reverse the inflammatory mediators caused by LPS by decreasing the expression of phospho‐STAT3, IL‐6, and TNF‐α. Liraglutide was able to do this by AMPK phosphorylation [116]. In another study assessing keratinocyte migration, liraglutide was found to induce their migration in HaCat cells by P13k/Akt mediation [117]. In another study, liraglutide was found to enhance keratinocyte function in diabetic mice via binding to unconventional myosin 1, then upregulating dedicator of cytokinesis 5 signalling, and ultimately promoting wound healing [118]. Studies that investigate the impact of GLP‐1RAs on keratinocyte migration are needed in humans, and specifically in DFUs; however, these preclinical data provide a rationale for a role for GLP‐1RAs in promoting keratinocyte migration and wound healing in DFUs (Table 2).

**TABLE 2:** Glucagon like peptide‐1 receptor agonists mediation of diabetic foot ulcer pathogenesis mechanisms.

Glucagon like peptide‐1 receptor agonist	Mechanism	Specific studies
Exenatide	Microvascular function	Increased flap survival in murine models [51] Increased microvascular density in murine models [51] Increased VEGF expression in murine models [51] Increased local skin perfusion, mediated by eNOS, when injected into forearm dermis [56]
	Neuropathic symptoms	Did not reduce prevalence of peripheral neuropathy [63] Promoted corneal nerve regeneration [65]
	Inflammation	Reduced IL‐6, IL‐1β, NF‐κB, TLR4, and TNF‐α in murine models [51] Reduce IFN‐γ, IL‐17, IL‐2, TNF‐β, IL‐6 and IL‐1β expression [98, 99, 103] Change macrophages to their anti‐inflammatory phenotype [100, 103] Reduced TNF‐α, TLR‐2, TLR‐4, IL‐1β, MCP‐1 and IL‐6^106,107^
Liraglutide	Microvascular function	Enhanced angiogenesis, regulated by Hif‐1a‐heme oxygenase‐1 axis, and by regulating AMPK for endothelial promotion in murine models [54] Increased local skin perfusion, mediated by endothelial NOS, when injected into forearm dermis [56]
	Neuropathic symptoms	Did not alter neuronal function [64] Reduced Il‐6, a neuro‐inflammatory component of peripheral neuropathy [64] Improved enteric nervous system functioning [66]
	Apoptosis	Greater cell viability of renal tubular cells [71], pancreatic islet cells [72] and cardiomyocytes [73]
	Weight loss	FDA approved treatment for weight loss [78]
	Oxidative stress	Reduced oxidative stress in SH‐5Y5Y cells [84] Increased superoxide dismutase activity in SH‐5Y5Y cells [84] Decreased ROS content in SH‐5Y5Y cells [84]
	Keratinocyte migration	Induced keratinocyte migration in HaCat cells by P13k/Akt mediation [117] Enhanced keratinocyte function in murine models by mediating unconventional myosin 1 and dedicator of cytokinesis 5 signalling [118]
Semaglutide	Weight loss	FDA approved treatment for weight loss [78]
	Inflammation	Decrease hsCRP levels [103, 109]
Tirzepatide	Weight loss	FDA approved treatment for weight loss [78]
Exendin‐4	Apoptosis	Increase expression of cell survival markers in neuroblastoma cells [74] Decrease pro‐apoptotic markers in neuroblastoma cells [74] Lower ROS defence markers in neuroblastoma cells [74] Lower levels of expressions of mitochondrial function associated genes and mitochondrial fission genes in neuroblastoma cells [74] Increased expression levels of regulators of mitochondrial homeostasis in neuroblastoma cells [74] Decreased levels of defective mitochondrial accumulates [74]
	Inflammation	Reduce IFN‐γ, IL‐17, IL‐2, TNF‐β, IL‐6 and IL‐1β expression [98, 99, 103]
GLP‐1 Exposure	Apoptosis	Induced expression of a mitochondrial protective gene and protein in hepatocytes of a murine model [70]
	Oxidative stress	Reduced oxidative stress in human umbilical vein endothelial cells [85]
	Inflammation	Increased IL‐10 levels and activated the STAT3 pathway [101, 102, 103]

## 4 Mitigating Complications and Recurrence

GLP‐1RAs may influence the underlying pathogenesis of DFUs while also reducing complications and recurrence. In the LEADER (Liraglutide Effect and Action in Diabetes: Evaluation of Cardiovascular Outcome Results) trial, the GLP‐1RA liraglutide was associated with a 35% reduction in the risk of LEAs related to diabetic foot ulcers compared to placebo [119]. Additionally, a more recent retrospective population‐based cohort study found that GLP‐1RAs were linked to a lower risk of LEA [120].

Statins, or HMG‐CoA (3‐hydroxy‐3‐methylglutaryl coenzyme A) reductase inhibitors, influence the wound healing cascade [121]. By lowering lipid levels, improving endothelial function, increasing NO levels, and reducing thrombosis, inflammation, and oxidative stress, statins contribute to better wound healing [121, 122, 123]. Several studies in diabetic rodent models have confirmed the beneficial effects of statins on wound healing, particularly with pravastatin [124] and simvastatin [125]. Although studies on the role of statins in human chronic wounds are more limited, a randomized trial of 13 patients with DFUs found that higher doses of oral atorvastatin promoted healing and reduced recurrence by lowering the inflammatory marker C‐reactive protein [123]. Additionally, a retrospective study of 139 patients with DFUs established a strong association between statin use and a six‐week wound size reduction [126]. The combination of statins and GLP‐1RAs may offer a synergistic effect on wound healing, potentially accelerating wound closure, enhancing tissue regeneration, and reducing complications associated with chronic diabetic wounds.

## 5 Potential Harms of GLP‐1RAs

There are several side effects and potential harms to consider when using GLP‐1RAs. In a systematic review assessing adverse effects of GLP‐1RA use, common gastrointestinal disorders included nausea, diarrhoea, vomiting, constipation, and abdominal pain. Common cardiovascular side effects include an increase in heart rate. Common side effects include injection site reactions, such as rash, erythema, and itching. Infection side effects include upper respiratory and urinary tract infections, nasopharyngitis, influenza, cystitis, and viral infection. Other side effects include headache and acute kidney injury [127]. When analysing the US Department of Veterans Affairs database and comparing patients who initiated GLP‐1RAs to those who initiated sulfonylureas, dipeptidyl peptidase 4 inhibitors, or sodium−glucose cotransporter‐2 inhibitors, patients using GLP‐1RAs were more likely to have an increased risk of gastrointestinal disorders, hypotension, syncope, arthritic disorders, nephrolithiasis, interstitial nephritis, and drug‐induced pancreatitis [128].

A notable adverse event of GLP‐1RA use is muscle mass loss. In a systematic review of 19 RCTs with 1345 participants assessing GLP‐1RA use in patients, those who used GLP‐1RAs experienced greater loss of lean body mass when compared to non‐users [129]. Loss of lean body mass puts patients at risk for sarcopenia, causing worsening physical abilities. In a study assessing lower extremity muscle weakness in 36 patients with diabetes compared to nondiabetic controls, those with diabetes and those with diabetes who had a history of ulceration had greater intrinsic and extrinsic muscle weakness when compared to controls. It has been reported that muscle weakness in patients with diabetes is associated with foot deformities, which puts patients at greater risk for DFUs [130]. However, some argue that GLP‐1RA use can improve skeletal muscle function, since diabetes is a risk factor for skeletal muscle loss, and GLP‐1RA use can help treat patients' diabetes. Moreover, GLP‐1RA use can improve blood flow to skeletal muscles and reduce ectopic fat deposition surrounding skeletal muscles, potentially improving skeletal muscle function [129]. Regardless, it is important to consider that GLP‐1RA use can contribute to lean body mass loss, potentially increasing the risk of ulcerations in some patients.

## 6 Systematic Review

### 6.1 Materials and Methods

A systematic review was conducted assessing the rate of diabetic foot complications (DFCs) in patients taking GLP‐1RAs in comparison to placebo or another antidiabetic medication. This systematic review has been registered in the International Prospective Register of Systematic Reviews (PROSPERO, registration number: CRD420251031038) and was conducted in accordance with PRISMA guidelines. PubMed and Embase were systematically searched, with the search timeframe being from the establishment of the database to February 2025. The search terms were: (Glucagon‐like peptide‐1 receptor agonists OR dulaglutide OR exenatide OR liraglutide OR lixisenatide OR semaglutide OR albiglutide) AND (side effects OR adverse effects OR adverse events OR diabetic foot ulcer). Only articles published in the English language were included. Inclusion criteria included randomized controlled trials, adult participants with DM, experimental group receiving a GLP‐1RA, control group receiving placebo or another antidiabetic medication, and studies reporting diabetic foot complications as an adverse event. Exclusion criteria included participants under the age of 18 and an inability to obtain data. For data extraction, two independent researchers reviewed each document. If there was disagreement, a third researcher helped resolve the disagreement. A data extraction table was developed, which included author, year of publication, study sites, interventions, study duration, follow‐up time, and rates of diabetic foot complication in both the control and experimental groups.

### 6.2 Results

**FIGURE 2:**
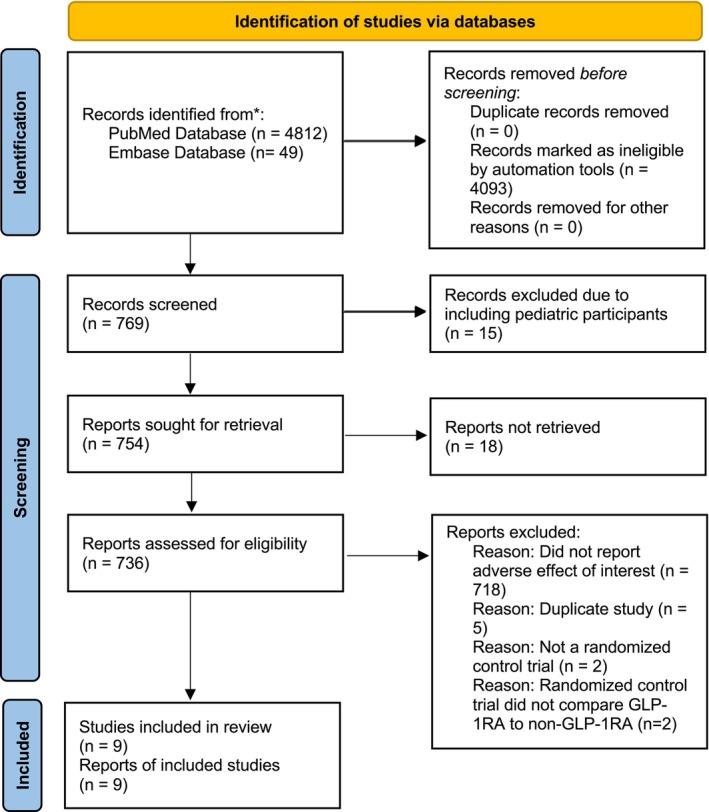
PRIMA flow diagram for the selection of studies for systematic review. This figure illustrates how records were selected to include in the systematic review.

We identified a total of 4812 records on PubMed and 49 on Embase. Automation tools determined 4093 records to be ineligible based on study design. We screened the titles and abstracts of the remaining 769 records (Figure 2). A total of nine records were included in the review, with a total of 14,110 participants and 39,450.3 patient‐years of data. The duration of follow‐up ranged from 0 weeks to 3.8 years. All but one of the records were multicenter studies [63, 131, 132, 133, 134, 135, 136, 137, 138]. Findings from the systematic review can be seen in Table 3.

**TABLE 3:** Results of adverse events by treatment group.

References	Experimental	Control	Intervention	Adverse event	Rate of adverse events in the experimental group	Rate of adverse events in the control group	Duration of follow‐up
Rockenstock et al. [131]	Tirzepatide + Insulin Glargine	Insulin Lispro + Insulin Glargine	Once weekly Tirzepatide (5 mg, 10 mg, or 15 mg) or prandial thrice‐daily Insulin Lispro (100 IU/mL) for 52 weeks	Diabetic Foot	1/717 = 0.14%	1/708 = 0.14%	4 weeks
				Diabetic Foot Infection	0/717 = 0%	2/708 = 0.28%	
D'Alessio et al. [132]	Liraglutide + Metformin ± Sulfonylurea	Insulin Glargine + Metformin ± Sulfonylurea	Once daily Liraglutide or Insulin Glargine plus Metformin ± Sulfonylurea for 24 weeks^a^	Diabetic Gangrene	1/484 = 0.21%	0/481 = 0%	N/A
Billings et al. [133]	Insulin Degludec/Liraglutide (IDegLira) + Metformin	Insulin Glargine (IGlar) + Insulin Aspart (IAsp) + Metformin	Once daily IDegLira or IGlar U100 (100 IU/mL) and IAsp (100 IU/mL) plus Metformin	Diabetic Foot Infection	1/252 = 0.40%	0/254 = 0%	4 weeks
				Diabetic Foot	0/252 = 0%	1/254 = 0.39%	
Blonde et al. [134]	Liraglutide + SGLT2i ± Metformin	Placebo	Once daily Liraglutide 1.8 mg or placebo for 26 weeks	Diabetic Foot Ulcer	0/202 = 0%	0/100 = 0%	1 week
Philis‐Tsimikas et al. [135]	Insulin Degludec/Liraglutide (IDegLira) + SGLT2i	Insulin Glargine (IGlar) + SGLT2i	Once daily IDegLira and IGlar U100 (100 IU/mL) plus SGLT2i	Infected Skin Ulcer	0/209 = 0%	1/210 = 0.005%	4 weeks
Marso et al. [136]	Liraglutide + SC^b^	Placebo + SC	Once daily Liraglutide 1.8 mg or placebo in addition to SC	Diabetic Foot Ulcer	181/4668 = 3.9%	198/4672 = 4.2%	3.8 years
Lingvay et al. [137]	Semaglutide + Metformin	Canagliflozin + Metformin	Once weekly Semaglutide 1.0 mg or Canagliflozin 300 mg PO daily for 52 weeks	Skin Ulcer	2/392 = 0.51%	0/394 = 0%	5 weeks
Pozzilli et al. [138]	Dulaglutide + Insulin Glargine ± Metformin	Placebo + Insulin Glargine ± Metformin	Once weekly Dulaglutide 1.5 mg or placebo for 28 weeks	Skin Ulcer	0/150 = 0%	1/150 = 0.7%	N/A
Jaiswal et al. [63]	Exenatide	Insulin Glargine	Twice daily Exenatide or daily Insulin Glargine for 18 months	Toe infection	1/22 = 4.5%	1/24 = 4.2%	18 months
				Plantar ulcer and cellulitis	0/22 = 0%	1/24 = 4.2%	

Abbreviation: GLP‐1 RA, glucagon‐like‐peptide‐1 receptor agonist.

^a^ 24‐week extension for uncontrolled liraglutide group.

^b^ Standard care (SC) is based on the LEADER standard care guidelines.

The occurrence of DFCs in this population is reportedly rare, potentially due to the limited follow‐up time available for observation in the included studies. Notably, Marso et al., who reported on 9340 participants and had the largest follow‐up time of 3.8 years, demonstrated that GLP‐1RAs do have a protective effect against DFCs, as they do not increase the risk of DFUs and have a lower risk of associated amputations (HR 0.65 [95% CI 0.45, 0.95; *p* = 0.03]) when compared to placebo [135]. A limitation of some of the included studies is the limited follow‐up time, which makes it challenging to assess if GLP‐1RAs truly mitigate DFU development. Additionally, many of the screened, but not included, studies reported an adverse event relating to the skin, but did not specify what type of skin‐related adverse event occurred, making it challenging to assess the true rate of DFCs. One notable study that did not meet the inclusion criteria, but is relevant to the discussion, is a retrospective cohort study utilizing the TriNetX US Research Network data looking at the effect of semaglutide on DFU outcomes when compared to semaglutide non‐users across 125,150 patients. This study found that at one year, patients taking semaglutide had statistically significant lower relative risks for wound healing complications, chronic non‐healing wounds, chronic pain, wound care, wound dehiscence, and amputation, with similar findings at five years [139]. Ultimately, further RCTs assessing the rate of DFCs in patients using GLP‐1RAs need to be completed.

## 7 Future Directions

To advance the therapeutic potential of GLP‐1RAs and dual or triple agonists, future research should prioritize clinical trials using these agents compared to standard of care in DFUs, as well as head‐to‐head studies assessing their impact on wound healing outcomes, including metrics such as wound closure rates, recurrence, and measures of skin barrier function like TEWL. TEWL measurement is not typically used in wound care to assess skin barrier restoration, but it has been applied in clinical studies for conditions like psoriasis and atopic dermatitis to gauge disease severity [140]. Incorporating TEWL measurement into wound care could provide a more comprehensive assessment of skin healing by not only tracking re‐epithelialization, but also evaluating the restoration of the skin's protective barrier, which is crucial for preventing infection and promoting long‐term healing. A multi‐centre non‐interventional study involving patients with diabetes including recently healed DFUs was conducted to evaluate whether TEWL is a reliable biomarker for predicting chronic wound recurrence, and they found that higher wound‐site TEWL scores were predictive of wound recurrence [141]. These results suggest that incorporating TEWL measurement into wound care could serve as a valuable tool for predicting chronic wound recurrence and improving long‐term healing outcomes.

Additionally, GLP‐1RAs have shown promise in modulating the unique physiology of plantar skin. Evidence from in vitro studies has demonstrated that GLP‐1RAs (such as liraglutide and exenatide) can enhance keratinocyte migration through PI3K/AKT and AMPK pathway activation, pathways that are impaired in diabetic skin [116]. These mechanistic effects may be particularly relevant to plantar skin, where re‐epithelialization is inherently delayed. As such, GLP‐1RAs may have a unique therapeutic potential in modifying the plantar skin environment, reducing mechanical fragility, improving keratinocyte turnover and barrier restoration, and thereby preventing DFU formation and recurrence. Furthermore, because plantar skin resembles a constitutively stressed or pre‐wounded state [45], its response to systemic metabolic and inflammatory cues may be particularly sensitive to GLP‐1RA‐mediated modulation. Future studies focusing on GLP‐1RAs' effects on plantar skin architecture, function, and recurrence metrics are warranted.

The following dual and triple agonists are currently in phase 3 clinical trials: Cagrilintide/Semaglutide, a GLP‐1/amylin dual agonist; Survodutide, a GLP‐1/glucagon dual agonist; Mazdutide, a GLP‐1/GIP dual agonist; and Retatrutide, a triple agonist [142]. Phase 2 data from Retatrutide clinical trials have shown promising results in terms of weight reduction and glycemic control [143, 144]. These emerging agents hold promise for advancing wound healing outcomes, with future studies needed to assess their efficacy. Moreover, the integration of statins into these trials could provide valuable insights into their potential synergistic effects, given their established benefits in reducing inflammation, improving endothelial function, and promoting tissue repair. Such comprehensive studies could help establish optimal dosing, administration strategies, and combination therapies to maximize efficacy in managing complications like chronic wounds.

Several oral GLP‐1RAs are currently under development for T2DM and obesity, with high doses of oral semaglutide (25 and 50 mg) demonstrating good tolerability and higher efficacy in weight loss and lower mean HbA1c levels compared to oral semaglutide 14 mg in phase 3 trials [145]. However, as of now, these doses have not received regulatory approval for clinical use. Orforglipron, an oral, non‐peptide, GLP‐1RA is also undergoing phase 3 trials as treatment for T2DM and obesity (NCT05872620) [146].

## 8 Conclusion

GLP‐1RAs are agents that have been used in patients with diabetes for many years, but their utility in managing DFUs is less well known. GLP‐1RAs modulate microvascular function, neuropathic symptoms, apoptosis, weight loss, oxidative stress, inflammation, and keratinocyte migration, which are all factors critical to DFU development, suggesting their potential in treating DFUs. DFUs are a burden to the healthcare system, not only because of their expense but also their burden to patients, resulting in preventable infections, hospitalizations, amputations, declining functional status, and even death [1]. More human studies assessing the efficacy of GLP‐1RAs in managing DFUs are essential, as these agents provide promise in treating a burdensome and preventable disease.

## Author Contributions

**Fiona S. Gruzmark:** conceptualization, writing – original draft. **Gabriela E. Beraja:** conceptualization, writing – original draft. **Ivan Jozic:** conceptualization, writing – review and editing. **Hadar A. Lev‐Tov:** conceptualization, writing – review and editing, supervision, project administration.

## Conflicts of Interest

The authors declare no conflicts of interest.

## Data Availability

The data that support the findings of this study are available on request from the corresponding author. The data are not publicly available due to privacy or ethical restrictions.
